# A cross-sectional survey on the early impact of COVID-19 on the uptake of decentralised trial methods in the conduct of clinical trials

**DOI:** 10.1186/s13063-022-06706-x

**Published:** 2022-10-06

**Authors:** Arnela Suman, Jasmijn van Es, Helga Gardarsdottir, Diederick E. Grobbee, Kimberly Hawkins, Megan A. Heath, Isla S. Mackenzie, Ghislaine van Thiel, Mira G. P. Zuidgeest, on behalf of the Trials@Home Consortium

**Affiliations:** 1 Julius Center for Health Sciences and Primary Care, University Medical Center Utrecht, Utrecht, The Netherlands; 2 Amphia Academy, Amphia Hospital, Breda, The Netherlands; 3 Division of Pharmacoepidemiology and Clinical Pharmacology, Utrecht Institute for Pharmaceutical Sciences, Faculty of Science, Utrecht University, Utrecht, The Netherlands; 4 Sanofi-Aventis Recherche & Development, Chilly Mazarin, Île-de-France France; 5 MEMO Research, Division of Molecular and Clinical Medicine, University of Dundee, Dundee, UK

**Keywords:** Decentralised trial methods, Digital health solutions, Decentralised clinical trials, COVID-19, Clinical trials

## Abstract

**Background:**

The COVID-19 pandemic significantly impacted the conduct of clinical trials through delay, interruption or cancellation. Decentralised methods in clinical trials could help to continue trials during a pandemic. This paper presents the results of an exploratory study conducted early in the pandemic to gain insight into and describe the experiences of organisations involved in clinical trials, with regard to the impact of COVID-19 on the conduct of trials, and the adoption of decentralised methods prior to, and as mitigation for the impact, of COVID-19.

**Methods:**

A survey with 11 open-ended and four multiple choice questions was conducted in June 2020 among member organisations of the public-private “Trials@Home” consortium. The survey investigated (1) the impact and challenges of COVID-19 on the continuation of ongoing clinical trials, (2) the adoption of decentralised methods in clinical trials prior to and as a mitigation strategy for COVID-19, (3) the challenges of conducting clinical trials during COVID-19, (4) the expected permanency of COVID-19-driven changes to the adoption of decentralised methods in clinical trials, and (5) lessons learned from conducting clinical trials during the COVID-19 pandemic. A thematic, inductive analysis of open survey questions was performed, complemented with descriptive statistics (frequencies and distributions).

**Results:**

The survey had a response rate of 81%. All organisations included in the analysis (*n* = 18) implemented (some) decentralised methods in their clinical trials prior to COVID-19, and 15 (83%) implemented decentralised methods as mitigation for COVID-19. Decentralised methods for IMP supply, patient-health care provider interaction and communication, clinic visits and source document verification were used more often as mitigation strategies than they were used prior to COVID-19. Many respondents expect to maintain those decentralised methods they implemented during COVID-19 in ongoing trials, as well as implement them in future trials.

**Conclusions:**

Decentralised methods are a widely implemented mitigation strategy for trial conduct in the face of the COVID-19 pandemic. The results of this survey show that there is an interest to continue the use of decentralised methods in future trials, but important points of attention have been identified that need solutions to help guide the transition from the traditional trial model to a more decentralised trial model.

**Supplementary Information:**

The online version contains supplementary material available at 10.1186/s13063-022-06706-x.

## Background

The ongoing COVID-19 pandemic has significantly impacted all aspects of health care worldwide. Governments and health care providers implemented a number of strategies to limit transmission, prioritise deployment of health care professionals and protect the capacity of their health care systems [1]. Mitigation strategies such as prioritisation of medical and research staff and services to COVID-19-related clinical care, social distancing, reduced volume of public transportation and stay-at-home restrictions have resulted in deferred delivery of health services, delay or avoidance of medical care and disruptions in the conduct of clinical trials [2–6]. Due to COVID-19, clinical trials have faced numerous challenges to the continuation of various trial elements, including recruitment and enrolment of new participants/patients, follow-up and monitoring of participants, outcome measurements and delivery and administration of (investigational) drugs and devices [6–10]. The disruption of clinical trial delivery has obvious negative consequences for the development of novel or improved therapies for patients and the delivery of care to trial participants in these trials. Additionally, discontinuation of ongoing trials leads to a morally and ethically unacceptable resource waste, both from participants’ and investigators’ efforts and resource perspectives [11].

Implementing decentralised methods for clinical trials can be used to safeguard the continuation of clinical trials and to oversee participants’ care during COVID-19. Studies show that for instance, recruitment, enrolment, follow-up and monitoring of participants have been converted to telephone and telemedicine visits where appropriate; standardised telephone interviews and use of smartphone apps have been encouraged for outcome measurements, and investigational medicinal products (IMPs) have been distributed directly to participants’ homes to limit infection transmission risk and comply with local regulations and restrictions during the pandemic [7, 9, 12, 13]. Several of these methods and digital innovations are key to the concept of decentralised clinical trials (DCTs). DCTs are clinical trials that make use of digital innovations and other related methods to make them more accessible to participants and reduce the burden of attending a clinical trial site [14]. DCTs can be hybrid trials that use only limited decentralised methods in combination with more conventional site-based methods, as well as fully “virtual” or “digital” trials where there may be no direct interaction between study personnel and participants and where visits to a clinical trial centre are minimised or eliminated and moved to the participants’ direct surroundings [14]. DCTs may potentially reduce participant burden, accelerate the recruitment process, increase enrolment and diversity of participants and reduce the number of investigator sites and research staff needed [15–20]. Retention rates may be positively influenced by this reduced participant burden and increased participant engagement through web-based platforms [15–19]. Despite the potential advantages of decentralised trial methods and digital health solutions, the adoption of DCT methodology has been slow up until the COVID-19 pandemic [19–22]. The pandemic-induced first round of large-scale experience with decentralised methods in clinical trials may provide lessons for and anticipate future challenges and opportunities.

As COVID-19 increased interest in and application of decentralised trial methods, an exploratory survey on the uptake of decentralised trial methods in the early phase of the pandemic was conducted among member organisations of the “Trials@Home” research consortium, a public-private partnership funded through the Innovative Medicines Initiative, with the aim to develop recommendations and tools for the definition and operationalisation of DCTs in Europe.

The aim of this survey, carried out early in the pandemic, was to gain insight into and describe the experiences of the consortium member organisations with regard to the impact of COVID-19 on the conduct of ongoing trials and the adoption of decentralised methods prior to COVID-19 and as a mitigation for the impact of COVID-19 on ongoing clinical trials.

## Methods

The reporting of this survey study follows the guidance provided by the “good practice in the conduct and reporting of survey research” paper [23].

### Development of the survey

**Fig. 1:**
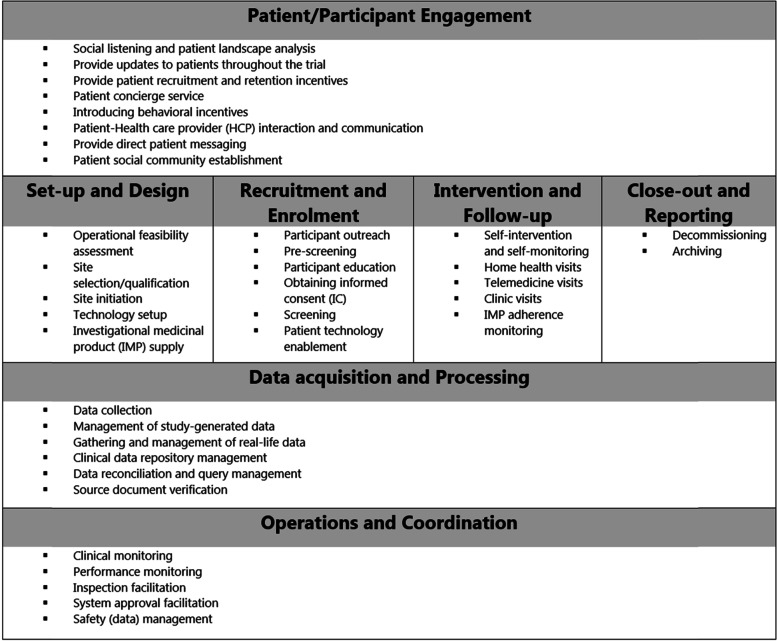
Basic building blocks and common trial activities within these blocks

An electronic survey, consisting of 11 open-ended questions and four multiple-choice questions, was developed in Microsoft Word. Ongoing work performed in Trials@Home to gain insight into the current best practices with regard to decentralised methods in clinical trials, and a survey published by the American Society of Clinical Oncology on the early effects of COVID-19 on clinical trials informed the design of the survey [12, 24, 25]. To facilitate thinking about the various stages of a clinical trial in which decentralised methods can be implemented, the *basic building blocks* (BBB) approach that is used in the Trials@Home consortium was used to guide the multiple-choice questions in this survey. The BBB approach consists of 7 high-level trial building blocks, and each block can be further broken down into specific trial activities for which decentralised methods can be adopted. Figure 1 shows these high-level trial building blocks and provides a list of common trial activities for each building block. This is not an exhaustive list, and a more detailed description of the BBB approach, definitions and activities has been published elsewhere [26]. The survey was reviewed for textual defects, clarity and ethical formulation and the omission of any relevant topics by a core team consisting of researchers, epidemiologists, trial operational experts and ethicists. Subsequently, the survey was piloted by three Trials@Home member organisations, i.e. a contract research organisation (CRO), a university and a pharmaceutical company.

### Outcomes

The following are the outcomes of primary focus:

1. How COVID-19 impacted the conduct of clinical trials in the organisations, i.e. trials continued with modifications (including the changes made for these trials to continue such as adoption of decentralised methods), trials continued without modification (including the main characteristics of these trials) and trials were put on hold (including reasons for discontinuing these trials)
2. The uptake of decentralised trial methods (including the type of activities conducted remotely) and the conduct of fully decentralised trials prior to COVID-19
3. The uptake of decentralised trial methods (including the type of activities conducted decentralised) and the conduct of fully decentralised trials as a mitigation for COVID-19

The following are the outcomes of secondary focus:

1. The challenges to trial conduct posed by COVID-19
2. Decentralised methods that did or did not work well, including reasons
3. The decentralised methods expected and planned to be maintained after COVID-19 in ongoing or future trials
4. Important lessons learned from COVID-19 on clinical trial conduct

The following are the other outcomes:

1. The responding organisation’s usual role in clinical trials (e.g. industry sponsor, CRO)
2. The response to the survey being on behalf of the entire organisation or one unit/department within the organisation
3. The number of ongoing clinical trials prior to COVID-19

### Participants

Trials@Home (www.trialsathome.com) is a public-private partnership consisting of a consortium of 32 organisations. Trials@Home aims to reshape clinical trial design, conduct and operations, by developing and piloting standards, recommendations and tools for the definition and operationalisation of DCTs in Europe. The consortium covers various types of organisations in non-profit and profit sectors, i.e. universities, university medical centres, research networks, patient organisations, CROs, pharmaceutical and technology companies and a medical law consultancy firm. The member organisations are located (at least partly) in Europe, but operating worldwide. No incentives for participation in this survey were provided.

### Data collection and analysis

The electronic survey was sent out via personal e-mails to all Trials@Home consortium member organisations in June 2020, with subsequent reminders sent after a week and another after 2 weeks. All data received from the survey were entered into a Microsoft Excel database for analysis by two researchers. To ensure the quality of the data, one researcher entered all data into the database, and the second researcher cross-checked all data entries for incorrectly entered, missing or redundant data [27].

To organise, describe and interpret the experiences of the survey respondents, a thematic analysis with an inductive approach was used to analyse the data from the open survey questions [28]. Using this approach, data were grouped into themes, which were subsequently analysed quantitatively, i.e. the frequencies and distributions of the themes were mapped and presented. Frequencies and distributions were also analysed for the multiple-choice survey questions.

### Data confidentiality

To secure the confidentiality of the data, the survey responses were stored using a unique identifier for each organisation. The data are stored securely at the University Medical Centre Utrecht (UMCU), where access is limited to the Trials@Home UMCU study team. The survey responses are pseudonymised and aggregated to ensure objective data analysis and presentation of the results and to decrease the possibility of information being traced back to individuals or organisations. Participation in the survey was voluntary, and consequently, consent to use the survey data was implied by filling out the survey questionnaire.

## Results

The survey was sent to all 32 member organisations, of which 26 responded to the survey, resulting in a response rate of 81%. Of these responses, 18 were included in the analysis. The remaining survey responses were excluded because the respective organisations were not directly involved in clinical trials (*n* = 6), did not systematically gather information necessary to complete the survey (*n* = 1) or did not send in data (*n* = 1). Not all survey questions were relevant to all organisations; therefore, no surveys were excluded due to incompleteness. The included responses originated from CROs (*n* = 2), pharmaceutical companies (*n* = 9), research networks (*n* = 2), technology companies (*n* = 3) and universities (*n* = 2). Table 1 provides an overview of the respondents’ characteristics. Six member organisations did not respond to the survey, of which 1 CRO, 2 pharmaceutical companies, 1 research network and 2 universities.

**Table 1:** Descriptive characteristics of survey respondents (*n* = 18)

Organisation type and number of ongoing clinical trials prior to COVID-19	Organisation’s usual role in clinical trials	Reply on behalf of	Geographical location
**CRO (*n*** **= 2)**			
**≥ 100**	CRO	Organisation	USA/Europe
**10 to < 100**	CRO, SMO	Organisation	Europe
**Pharmaceutical company (*n*** **= 9)**			
**≥ 100**	Industry sponsor	Organisation	Europe/UK
	Industry sponsor	Organisation	Others
	Industry sponsor	Organisation	USA
	Industry sponsor	Organisation	Europe
**10 to < 100**	Industry sponsor	Organisation	Europe
	Industry sponsor	Unit/department	USA/Europe
	Industry sponsor	Organisation	Others
	Industry sponsor	Organisation	Europe
**Unknown**	Industry sponsor	Organisation	Europe
**Research Network (*n*** **= 2)**			
**10 to < 100**	Technology provider	Unit/department	Europe
**< 10**	Site	Unit/department	Europe
**Technology company (*n*** **= 3)**			
**10 to < 100**	Technology provider	Organisation	UK
	Technology provider	Unit/department	Europe
**< 10**	Technology provider	Unknown	Europe
**University (*n*** **= 2)**			
**< 10**	Clinical Trial Unit	Unit/department	Europe
	Clinical Trial Unit	Unit/department	UK

### The impact and challenges of COVID-19 on the continuation of ongoing clinical trials

When asked how the COVID-19 pandemic impacted the continuation of clinical trials (continuation without modifications vs. with modifications vs. halting of trials), almost all respondents reported a combination of these three options. Table 2 shows the impact of COVID-19 on the continuation of ongoing trials.

**Table 2:** Impact of COVID-19 on the continuation of ongoing clinical trials

Trials continued with modifications		Trials continued without modifications		Trials put on hold	
***N*** of organisations (%)	Proportion of trials	***N*** of organisations (%)	Proportion of trials	***N*** of organisations (%)	Proportion of trials
2 (11)	None	1 (6)	None	2 (11)	None
4 (22)	1–25%	9 (50)	1–25%	7 (39)	1–25%
3 (17)	26–50%	6 (33)	26–50%	5 (28)	26–50%
4 (22)	51–75%	1 (6)	51–75%	2 (11)	51–75%
5 (28)	76–99%	0 (0)	76–99%	2 (11)	76–99%
0 (0)	100%	1 (6)	100%	0 (0)	100%

Seventeen respondents (94%) reported having continued a proportion of their trials without modifications. The respondents indicated that the main characteristics of these trials were related to the design, e.g. trial designs that had already implemented many decentralised aspects (53%, *n* = 9); the trial stage, e.g. trials had entered long-term follow-up or close-out stage (47%, *n* = 8); and trials where little or no patient encounters or visits were necessary or remaining (29%, *n* = 5). Other characteristics included trials with populations with a high medical need (24%, *n* = 4) and trials without IMP concerns, e.g. IMP had already been dispensed or delivery and administration raised no concerns (17%, *n* = 3). Less frequently reported characteristics were trials where no source document verification (SDV) was required (12%, *n* = 2), trials where established medication with known risk benefit profile was used (6%, *n* = 1) and trials that were conducted in regions unaffected by COVID-19 (6%, *n* = 1).

Sixteen respondents (89%) reported being able to continue a proportion of their ongoing trials with modifications. Modifications to ongoing trials included the implementation of decentralised methods, changing to fully decentralised operations or other modifications. Other modifications to trials included delay of study start/execution (13%, *n* = 2), (temporary) halt of inclusion (13%, *n* = 2), reviewing photos for diagnostics instead of in-person visits (6%, *n* = 1), postponing trial assessments (6%, *n* = 1) and adjusting trial sample size (6%, *n* = 1). Trials that continued with modifications during COVID-19 covered a broad range of therapeutic areas, but the therapeutic areas in which trials continued with the modification that were mentioned by the responding organisations most often included oncology (in 8 organisations), cardiovascular disorders (in 6 organisations) and neurology (in 6 organisations).

Sixteen respondents (89%) reported that they had to put a proportion of their trials on hold. Reported reasons for discontinuation of ongoing trials were mainly safety concerns for patients and staff (44%, *n* = 7) and closure of facilities (e.g. lab, sites, deliveries) due to lockdown measures (31%, *n* = 5), followed by restrictions to in-person visits (25%, *n* = 4) and avoiding unnecessary exposure (19%, *n* = 3). Less frequently reported reasons were lack of staff availability at sites (13%, *n* = 2), sites not accepting patients (6%, *n* = 1) and travel restrictions (6%, *n* = 1).

### The adoption of decentralised methods in clinical trials prior to and as a mitigation strategy for COVID-19

**Fig. 2:**
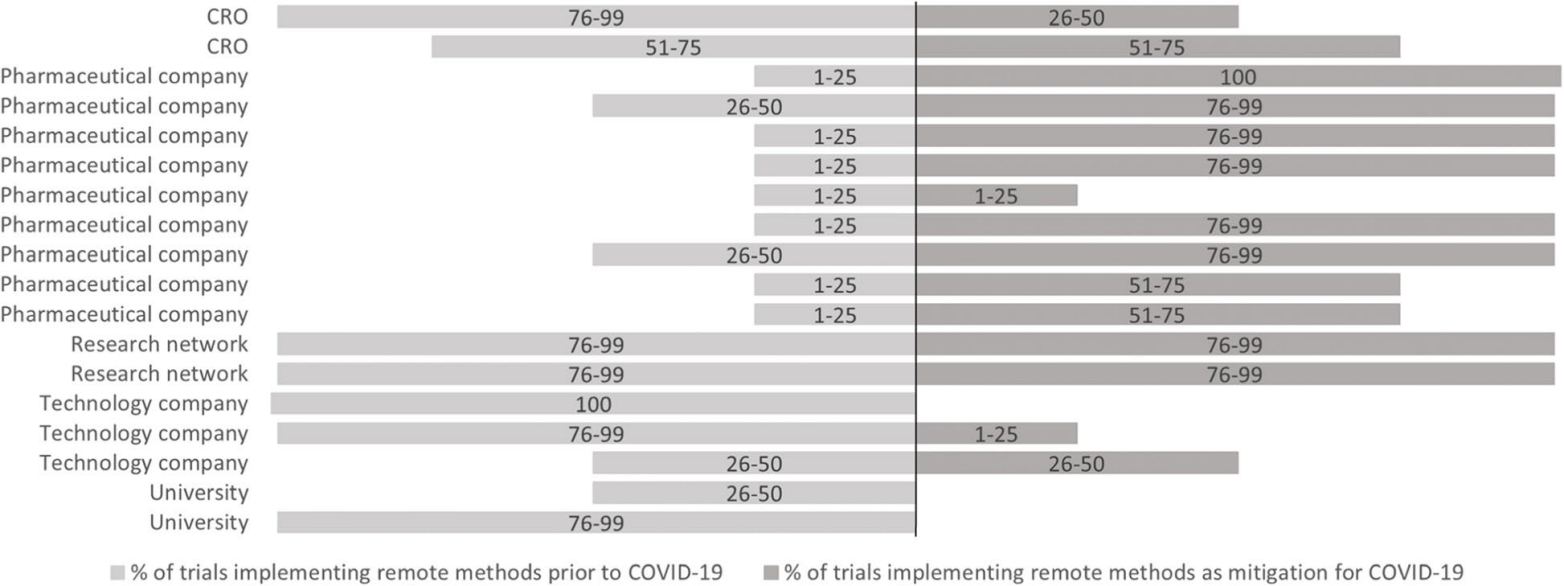
Percentage of trials in which some decentralised methods are implemented (not completely decentralised trials). *Respondents that did not answer this question are left blank in figure

**Fig. 3:**
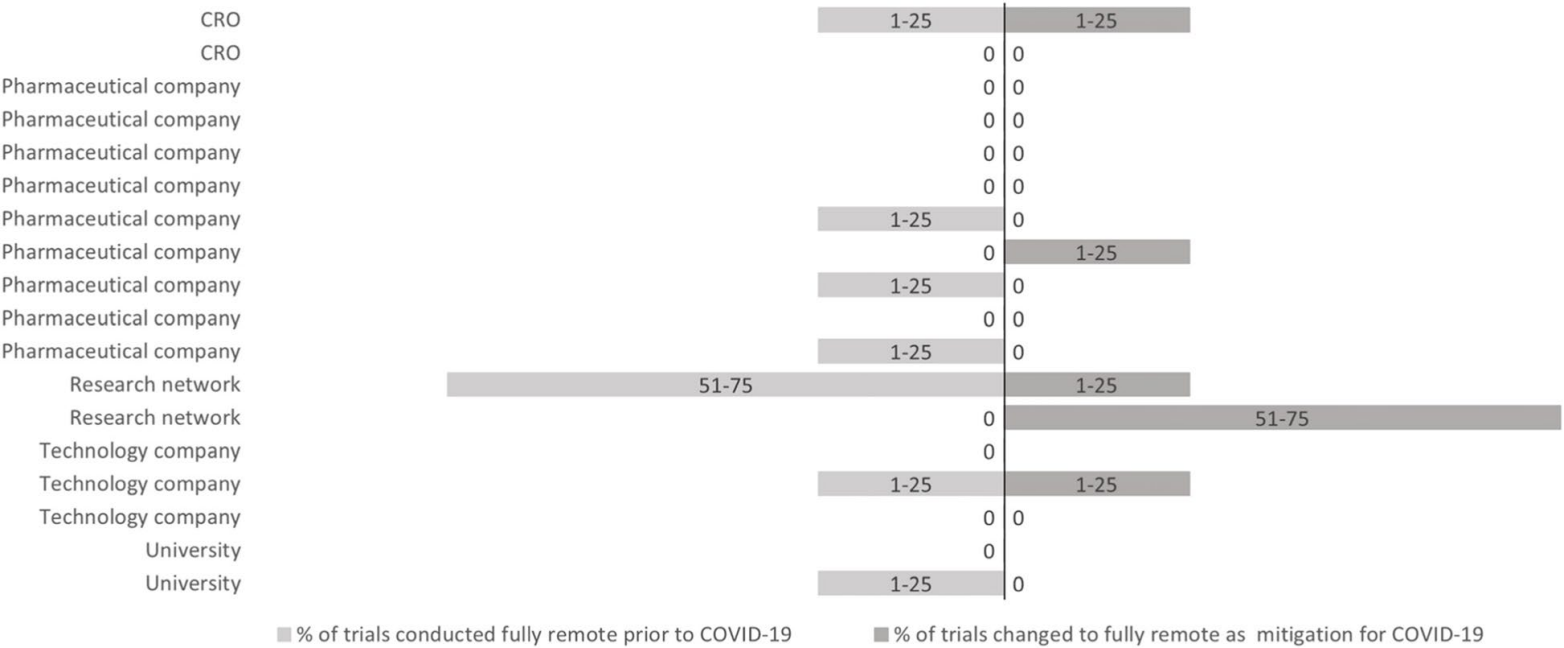
Percentage of trials that were conducted fully decentralised prior to COVID-19, and percentage of trials that changed to fully decentralised as a consequence of COVID-19. *Respondents that did not answer this question are left blank in figure

Seventeen respondents (94%) reported that a proportion of the clinical trials that were ongoing in their organisations prior to COVID-19 implemented decentralised methods. One respondent (6%) reported all of their organisation’s ongoing clinical trials prior to COVID-19 already implementing decentralised methods. Fifteen respondents (83%) reported that their organisation implemented decentralised methods in their clinical trials as a mitigation strategy for COVID-19. Figure 2 shows the percentage of trials implementing decentralised methods prior to and as a mitigation for COVID-19, per organisation. Figure 3 shows the percentage of trials that were conducted fully decentralised. Seven respondents (39%) reported a proportion of their organisations’ ongoing clinical trials prior to COVID-19 being fully decentralised. Five respondents (31%) reported that a proportion of their organisations’ clinical trials turned into fully decentralised trials in order to be able to continue during COVID-19. Of these, 2 respondents had no fully decentralised trials prior to COVID-19.

Prior to COVID-19, organisations most often adopted decentralised methods for study activities in the BBB patient engagement and data acquisition and processing, followed by recruitment and enrolment, and intervention and follow-up (Table 3). The activities mostly mitigated with decentralised methods during COVID-19 were, as shown in Table 3, in the BBB: set-up and design, intervention and follow-up, operations and coordination and other trial activities. When zooming in on specific trial activities, more organisations used decentralised methods as mitigation, compared to use before COVID-19, for the following 4 specific trial activities: patient-health care provider interaction and communication, direct-to-patient IMP supply, clinic visits changed to telemedicine visits and source document verification. Table 3 shows the detailed results on the adoption of decentralised methods for BBB and trial activities in respondents’ clinical trials prior to and as a mitigation strategy for COVID-19.

**Table 3:** The adoption of decentralised methods prior to and as a mitigation strategy for COVID-19^a^

BBB and trial activities implementing decentralised methods	Prior to COVID-19, ***N*** respondents (%)^**c**^ (***n*** = 18 total)	As mitigation for COVID-19^**b**^, ***N*** respondents (%)^**c**^ (***n*** = 15 total)
***Patient/participant engagement***	*17 (94)*	*10 (67)*
Social listening and patient landscape analysis	9 (53)	3 (30)
Provide updates to patients throughout the trial	7 (41)	5 (50)
Provide patient recruitment and retention incentives	8 (47)	5 (50)
Patient concierge service	6 (35)	3 (30)
Introducing behavioural incentives	3 (18)	3 (30)
Patient-health care provider (HCP) interaction and communication	6 (35)	**7 (70)**
Provide direct patient messaging	6 (35)	3 (30)
Patient social community establishment	1 (6)	1 (10)
***Set-up and design***	*13 (72)*	***15 (100)***
Operational feasibility assessment	9 (69)	6 (40)
Site selection/qualification	12 (92)	11 (73)
Site initiation	9 (69)	9 (60)
Technology set-up	10 (77)	6 (40)
IMP supply	6 (46)	**10 (67)**
***Recruitment and enrolment***	*15 (83)*	*9 (60)*
Participant outreach	10 (67)	7 (78)
Pre-screening	11 (73)	6 (67)
Participant education	10 (67)	8 (89)
Obtaining informed consent	10 (67)	4 (57)
Screening	5 (33)	1 (11)
Patient technology enablement	6 (40)	6 (67)
***Intervention and follow-up***	*15 (83)*	***14 (93)***
Self-intervention and self-monitoring	8 (53)	6 (43)
Home health visits	12 (80)	7 (50)
Telemedicine visits	11 (73)	11 (79)
Clinic visits	6 (40)	**7 (50)**
IMP adherence monitoring	10 (67)	7 (50)
***Close-out and reporting***	*4 (22)*	*2 (13)*
Decommissioning	4 (100)	2 (100)
Archiving	2 (50)	2 (100)
***Data acquisition and processing***	*17 (94)*	*14 (93)*
Data collection	16 (94)	9 (64)
Management of study-generated data	13 (77)	6 (43)
Gathering and management of real-life data	12 (67)	6 (43)
Clinical data repository management	10 (59)	6 (43)
Data reconciliation and query management	13 (77)	6 (43)
Source document verification	7 (41)	**8 (57)**
***Operations and coordination***	*13 (72)*	***11 (73)***
Clinical monitoring	11 (85)	10 (91)
Performance monitoring	12 (92)	7 (64)
Inspection facilitation	5 (39)	5 (46)
System approval facilitation	5 (39)	4 (36)
Safety (data) management	12 (92)	6 (55)
***Other trial activities***	*4 (22)*	***4 (27)***
Investigator payments	1 (25)	–
Meetings (e.g. investigator, safety, data monitoring, adjudication)	1 (25)	1 (25)
Maintenance and fault checks of remote equipment	1 (25)	–
Patient panels and focus groups	1 (25)	–
Record linkage to national health and mortality databases	1 (25)	1 (25)
e-signatures	–	1 (25)
Remote close-down of (regional) study centres	–	1 (25)

^a^Not all respondents reported on each BBB; totals do not necessarily add up to 18/15

^b^Bold indicates these decentralised methods were more often implemented as mitigation for COVID-19 compared to their use prior to COVID-19

^c^Respondents per BBB as the proportion of total respondents; percentages for trial activities as the proportion of respondents for the specific BBB

### The challenges for conducting clinical trials during COVID-19

Seventeen respondents (94%) reported challenges in conducting clinical trials since the COVID-19 pandemic. The closure of facilities due to lockdown was reported most frequently (53%, *n* = 9). A lack of site staff availability and restrictions on in-person visits were both reported by 5 respondents (29%). Safety concerns for the staff and patients were reported by 4 respondents (24%), as were regulatory guidance and policies, i.e. not all decentralised methods and activities being accepted by regulators (frequently reported example being decentralised SDV), variety in (inter)national regulations and (institutional) policies and staying up to date with and acting in a timely manner on the changing regulatory landscape. Scaling of decentralised methods that had only been used as a pilot before and designing and implementing decentralised methods for many trials simultaneously was another challenge reported by 3 respondents (18%). Equally mentioned were conducting (remote) monitoring activities (18%, *n* = 3), especially in changing regulatory landscapes, delivery and administration of IMP to patients in the face of closure of facilities (18%, *n* = 3), and issues with regard to data (18%, *n* = 3). Data issues included data verification and privacy, missing (critical) data, data privacy regulations and policies, access to IT systems, and delays in data collection in various organisations. The burden on the sites due to introducing remote monitoring and virtual interactions, maintaining the initial study protocol and recruitment and retention of patients were all reported by 2 respondents (12%). Maintaining oversight, conducting remote SDV and participant support and training and changing the mindset of all parties involved were reported as challenges by 1 respondent each (6%).

Twelve respondents (67%) reported specific decentralised methods that did not work well in the early phase of the pandemic. Most frequently reported were remote SDV (42%, *n* = 5), due to the nature of the data or due to regulatory restrictions and guidance on, and ethical acceptance of remote SDV. Home health visits were reported by 3 respondents (25%), being difficult to implement due to long timelines to set up or due to patients not accepting home health nurses in their homes. Remote monitoring, remote data collection (endpoint assessment), decentralised training and eConsent were each reported by 1 respondent (8%).

### The expected permanency of COVID-19-driven changes to the adoption of decentralised methods in clinical trials

Fifteen respondents (83%) reported that their organisations are expecting or planning to maintain (some) decentralised methods in the current, ongoing trials after COVID-19. Decentralised methods that are expected to remain included telemedicine/home health visits (87%, *n* = 13); direct-to-patient IMP supply (53%, *n* = 8); decentralised data collection (33%, *n* = 5); remote monitoring (27%, *n* = 4); remote site selection, initiation and close-down (20%, *n* = 3); and remote support and training (7%, *n* = 1). These methods, complemented with remote patient recruitment (7%, *n* = 1) and remote SDV (7%, *n* = 1), were also mentioned as the methods that worked well during COVID-19. In general, decentralised methods for which implementation timelines were short, for which digital solutions and technologies were already well established and implemented, and for which operating procedures and vendors already existed were reported to have worked well in the early phase of the pandemic.

Eleven respondents (61%) reported that permanent changes to trial conduct were planned for future trials. Decentralised methods that are planned for future trials were mostly the ones that were reported to have worked well during COVID-19 and are expected to be maintained in the current, ongoing trials. Planned changes for future trials included direct-to-patient IMP supply (36 %, *n* = 4), telemedicine/home health visits (27%, *n* = 3), decentralised data collection (27%, *n* = 3), remote monitoring (27%, *n* = 3), eConsent (18%, *n* = 2), remote support and training (9%, *n* = 1) and eSignatures for contracts (9%, *n* = 1).

### Lessons learned from COVID-19 on clinical trial conduct

When asked about the most important lessons learned from COVID-19 with regard to clinical trial conduct, the possibility of decentralised methods making trials crisis resistant and allowing for the continuation of trials was most often reported (33%, *n* = 6). Many decentralised methods could be implemented in a timely manner, except for remote SDV and some types of physical patient assessments. Three respondents (17%) reported COVID-19 as pushing change towards more acceptance of decentralised methods and the development and implementation of business continuity plans. Proactive and quick regulatory guidance was considered a facilitator for the implementation of decentralised methods (11%, *n* = 2), as well as patient and staff flexibility in and support for adapting to changes (6%, *n* = 1). It was noted that trial participant safety and data integrity remain a point of attention (6%, *n* = 1) and that there is no “one-size-fits-all” decentralised approach that fits all studies and complies with all countries’ regulatory landscapes (6%, *n* = 1).

## Discussion

This study reports the results of a survey on COVID-19-related challenges to clinical trial conduct, and the adoption of decentralised methods to mitigate these challenges by member organisations of the Trials@Home consortium, in the early phase of the COVID-19 pandemic (June 2020). The survey showed that all responding organisations experienced an impact of COVID-19 on trial conduct, with 88.9% of organisations having to discontinue a proportion of their ongoing trials. In 88.9% of the responding organisations, other trials could continue with modifications, of which decentralised methods were adopted in 83% of organisations. In 28% of organisations, trials were changed to fully decentralised trials to be able to continue during COVID-19.

The building blocks and trial activities, for which decentralised methods were implemented as mitigation, were not necessarily the trial activities that were already conducted decentralised prior to COVID-19. Direct-to-patient IMP supply, patient-health care provider interaction and communication, clinic visits and SDV were used more as a mitigation strategy compared to their use prior to COVID-19. While DCTs hold the potential of making clinical trial delivery more resilient and inclusive, the question is how this potential can be fully realised. In the current survey, trials that were able to continue without modifications during COVID-19 were the ones that not only had designs that already included some decentralised methods, but also ones that were in a trial stage where no or few patient encounters were necessary or remaining (i.e. close-out stage). It appears challenging to implement decentralised methods in trial stages where there are still patient encounters remaining, and future research should focus on driving solutions forward for trial(s) (stages) with such interactions, making trials even more patient-centred in the future. To further aid in patient centricity, potential downsides of decentralised methods, e.g. reliance on electronic devices, required digital literacy skills and access to the internet, need to be investigated and accounted for.

In addition to the expected adoption of decentralised methods as a mitigation strategy for COVID-19, there are some building blocks and trial activities that were less often mitigated by decentralised methods. This was true for trial activities regarding patient/participant engagement, recruitment and enrolment, and intervention and follow-up. More specifically, when looking at particular trial activities, it appears that trial activities involving patient encounters were less often mitigated by decentralised methods, e.g. home health visits, pre-screening and obtaining informed consent than they were adopted in general before the pandemic. This trend seems counter-intuitive but does not indicate a downward trend in the use of these decentralised methods but rather their limited use for mitigation, which can often be explained by the specifics of the COVID-19 pandemic, e.g. home health visits are not a preferred decentralised method during large-scale lockdowns and limited face-to-face interactions to prevent infection transmission. Additionally, 89% of respondents reported that they had to put a proportion of their trials on hold, which is expected to be easier before enrolment has started than when participants already receive the intervention. This can explain why decentralised methods for recruitment and enrolment and informed consent trial activities were less often used as mitigation strategies during COVID-19.

An interesting challenge to the continuation of trials during the pandemic appeared to be the scaling of decentralised methods that were already in use. Decentralised methods that were expected to remain after the pandemic were those methods that were already in use prior to COVID-19, proved to be working well, for which digital solutions and technologies were already well-established and in use by the organisation, and for which operating procedures were in place and vendors contracted. If the reported and expected change is in the increase of what has already been done before, then the question arises to what extent the current pandemic has been a catalyst for innovation, rather than simply amplifying existing methods and practices. The survey results indeed show a large uptake of tried and tested decentralised methods, but little shift in the uptake of innovative, fully decentralised trials to mitigate the impact of COVID-19, as shown by the low number of respondents who implemented fully decentralised trials as a consequence of COVID-19 (*n* = 5). It is worthwhile investigating barriers and facilitators for the implementation and maintenance of new methods and large-scale innovation.

Sixteen respondents (89%) reported that they had to discontinue a proportion of their trials as a consequence of COVID-19, with over half of these respondents (*n* = 9) discontinuing over 25% of their trials. The discontinuation appeared to be mainly due to safety concerns for patients and staff and to the consequences of government responses to the pandemic. Similar results and challenges have been found by an American Society of Clinical Oncology (ASCO) survey on the early impact of COVID-19 on oncology trial conduct, where nearly 60% of reported trials suspended enrolment and ceased research-only visits [12]. Facing the future and making trials more crisis resistant will require all parties involved in clinical trials to develop procedures and methods that allow evaluation and assurance of patient safety without or with limited face-to-face interactions. Regulatory guidance so far has been built on the premise of physical evaluation of safety events by qualified physicians [28]. Therefore, health authorities and policymakers play an important role in the development and deployment of these procedures and should incorporate the valuable lessons learned during COVID-19 to move guidance permanently forward. Besides assuring trial continuation, innovations in this field can allow for the recruitment of patients who would normally not participate in trials due to geographical area, mobility or financial issues, further allowing for more diverse and generalisable patient populations and data and inclusion of rare diseases [29–32]. The impact of using more decentralised methods on the patient experience of participation and the challenges for investigators and staff deserve further exploration in future research.

Beyond the evident barriers to conducting (certain) trial activities decentralised reported in this survey, such as regulatory restrictions (e.g. e-Consent not permitted in all countries), and practical considerations (e.g. certain necessary physical assessments), other aspects were reported to hamper the implementation of decentralised methods and several areas require continued focus and development. One aspect interesting to highlight here relates to the data collected remotely. Data integrity and validity should always remain a point of attention, but may be especially important for data collected unsupervised by participants using (remote) digital technologies [24]. Patient privacy and data confidentiality and protection should remain points of attention through various trial activities, from direct-to-patient IMP supply to remote monitoring [33]. In this regard, remote SDV may be particularly challenging in light of direct remote access to electronic health records not normally being permitted. While regulatory innovations, such as USA’s Food and Drug Administration (FDA) Information Exchange and Data Transformation (INFORMED) Initiative [34], are enabling the use of virtually collected data in clinical trials, further guidance on what constitutes quality for virtually collected data is needed. Noteworthy is that the Trials@Home consortium is currently preparing interactions with health authorities on, among other topics, questions related to the data quality of remotely collected data.

### Strengths and limitations

This survey provides insight into the adoption of decentralised methods and the challenges for clinical trial conduct by non-profit and for-profit organisations. Including both types of organisations provides broad views and is a valuable addition to the current state of knowledge, as so far, mainly for-profit organisations have reported on the impact of COVID-19 on their trial conduct. While this survey provides a good insight, the results may underestimate the actual impact of COVID-19, as one-third of the responses were on behalf of a specific unit or department instead of the entire organisation. Furthermore, the timing of the survey, early in the pandemic, may have underestimated the impact of COVID-19 on trial conduct and the implementation of decentralised methods and may not have revealed the long-term impact of COVID-19. Regarding the methodological limitations of this study, the relatively small number of organisations in the survey, as well as the mainly open-ended questions in the survey, which require more interpretation than closed questions, should be mentioned. However, the participating organisation together account for a large number of clinical trials that were impacted during COVID-19, rendering valuable insights. Lastly, when interpreting the results, one should keep in mind that the survey respondents were organisations that are part of the Trials@Home consortium and selection bias might have influenced these results as these organisations are more likely to be interested in decentralised trial methods.

## Conclusions

Decentralised methods are a widely implemented mitigation strategy for trial conduct in the face of a pandemic, albeit not without challenges. The results of this survey show that there is an interest to continue the use of decentralised methods in future trials, but important points of attention have been identified that need solutions to help guide the transition from the traditional trial model to a more decentralised trial model.

## Supplementary Information

**Additional file 1.**

## Data Availability

The datasets used and/or analysed during the current study are available from the corresponding author upon reasonable request.

## Abbreviations

BBB: Basic building block
CRO: Contract research organisation
DCT: Decentralised clinical trial
FDA: Food and Drug Administration
IMPs: Investigational medicinal products
INFORMED: Information Exchange and Data Transformation Initiative
SDV: Source document verification
UMCU: University Medical Centre Utrecht

## References

[1] World Health Organization (2020). COVID-19 strategy update.

[2] International Monetary Fund (2020). Policy responses to COVID-19.

[3] Roser M, Ritchie H, Ortiz-Ospina E, Hasell J (2020). Coronavirus pandemic (COVID-19).

[4] World Health Organization (2020). The impact of the COVID-19 pandemic on noncommunicable disease resources and services: results of a rapid assessment.

[5] Czeisler MÉ, Marynak K, Clarke KE (2020). Delay or avoidance of medical care because of COVID-19–related concerns—United States, June 2020. MMWR Morb Mortal Wkly Rep.

[6] Bagiella E, Bhatt DL, Gaudino M (2020). The consequences of the COVID-19 pandemic on non-COVID-19 clinical trials. J Am Coll Cardiol.

[7] Meschia JF, Barrett KM, Brown RD, Turan TN, Howard VJ, Voeks JH (2020). The CREST-2 experience with the evolving challenges of COVID-19. A clinical trial in a pandemic. Neurology.

[8] Rusen ID (2020). Challenges in tuberculosis clinical trials in the face of the COVID-19 pandemic: a sponsor’s perspective. Trop Med Infect Dis.

[9] Anker SD, Butler J, Khan MS, Abraham WT, Bauersachs J, Bocchi E (2020). Conducting clinical trials in heart failure during (and after) the COVID-19 pandemic: an expert consensus position paper from the Heart Failure Association (HFA) of the European Society of Cardiology (ESC). Eur Heart J.

[10] Ledford H (2020). Coronavirus shuts down trials of drugs for multiple other diseases. Nature.

[11] Chan A, Song F, Vickers A, Jefferson T, Dickersin K, Gøtzsche PC (2014). Increasing value and reducing waste: addressing inaccessible research. Lancet.

[12] Waterhouse DM, Harvey RD, Hurley P, Levit LA, Kim ES, Klepin HD (2020). Early impact of COVID-19 on the conduct of oncology clinical trials and long-term opportunities for transformation: findings from an American Society of Clinical Oncology survey. JCO Oncol Pract.

[13] Upadhaya S, Yu JX, Oliva C, Hooton M, Hodge J, Hubbard-Lucey VM (2020). Impact of COVID-19 on oncology clinical trials. Nat Rev Drug Discov.

[14] Trials@Home WP2 – TECH (2020). D2.1 Glossary of terms and definitions used.

[15] Laggis CW, Williams VL, Yang X, Kovarik CL (2019). Research techniques made simple: teledermatology in clinical trials. J Invest Dermatol.

[16] Moseson H, Kumar S, Juusola JL (2020). Comparison of study samples recruited with virtual versus traditional recruitment methods. Contemp Clin Trials Commun.

[17] Ali Z, Zibert JR, Thomsen SF (2020). Virtual clinical trials: perspectives in dermatology. Dermatology.

[18] Rosa C, Campbell AN, Miele GM, Brunner M, Winstanley EL (2015). Using e-technologies in clinical trials. Contemp Clin Trials.

[19] Apostolaros M, Babaian D, Corneli A, Forrest A, Hamre G, Hewett J (2020). Legal, regulatory, and practical issues to consider when adopting decentralized clinical trials: recommendations from the Clinical Trials Transformation Initiative. Ther Innov Regul Sci.

[20] Perry B, Geoghegan C, Lin L, McGuire F, Nido V, Grabert BK (2019). Patient preferences for using mobile technologies in clinical trials. Contemp Clin Trials Commun.

[21] Coran P, Goldsack JC, Grandinetti CA, Bakker JP, Bolognese M, Dorsey ER (2019). Advancing the use of mobile technologies in clinical trials: recommendations from the Clinical Trials Transformation Initiative. Digit Biomark.

[22] Bakker JP, Goldsack JC, Clarke M, Coravos A, Geoghegan C, Godfrey A (2019). A systematic review of feasibility studies promoting the use of mobile technologies in clinical research. npj Digit Med.

[23] Kelley K, Clark B, Brown V, Sitzia J (2003). Good practice in the conduct and reporting of survey research. International J Qual Health Care.

[24] Trials@Home WP1 – BEST (2020). D1.1 First set of recommendations for RDCTs (to be implemented in the pan-EU pilot RDCT).

[25] Trials@Home WP1 – BEST (2020). D1.2 Criteria for selection of appropriate trials.

[26] Trials@Home WP2 – TECH (2020). D2.3 Technology scan.

[27] Braun V, Clarke V (2006). Using thematic analysis in psychology. Qual Res Psychol.

[28] Balevic S, Singler L, Randell R, Chung RJ, Lemmon ME, Hornik CP. Bringing research directly to families in the era of COVID-19. Pediatr Res. 2020; [ePub ahead of print]. 10.1038/s41390-020-01260-1 PMC7656893 33177676

[29] Schneider RB, Biglan KM (2017). The promise of telemedicine for chronic neurological disorders: the example of Parkinson’s disease. Lancet.

[30] Clinical Trials Transformation Initiative (2018). CTTI recommendations: decentralized clinical trials.

[31] Khozin S, Coravos A (2019). Decentralized trials in the age of real-world evidence and inclusivity in clinical investigations. Clin Pharmacol Therapeut.

[32] Marsch LA, Campbell A, Campbell C, Chen CH, Ertin E, Ghitza U (2020). The application of digital health to the assessment and treatment of substance use disorders: the past, current, and future role of the National Drug Abuse Treatment Clinical Trials Network. J Subst Abuse Treat.

[33] Tan AC, Ashley DM, Khasraw M (2020). Adapting to a pandemic—conducting oncology trials during the SARS-CoV-2 pandemic. Clin Cancer Res.

[34] Khozin S, Kim G, Pazdur R. From big data to smart data: FDA’s INFORMED initiative. Nat Rev Drug Discov. 2017;16(306). 10.1038/nrd.2017.26 28232724

