# Supplementary material for: A cross-sectional survey on the early impact of COVID-19 on the uptake of decentralised trial methods in the conduct of clinical trials
Source: Trials. 2022 Oct 6;23:856. doi: 10.1186/s13063-022-06706-x (PMC9535935; doi:10.1186/s13063-022-06706-x)
Supplement: Supplementary file 1 — Additional file 1. [file 13063_2022_6706_MOESM1_ESM.docx]

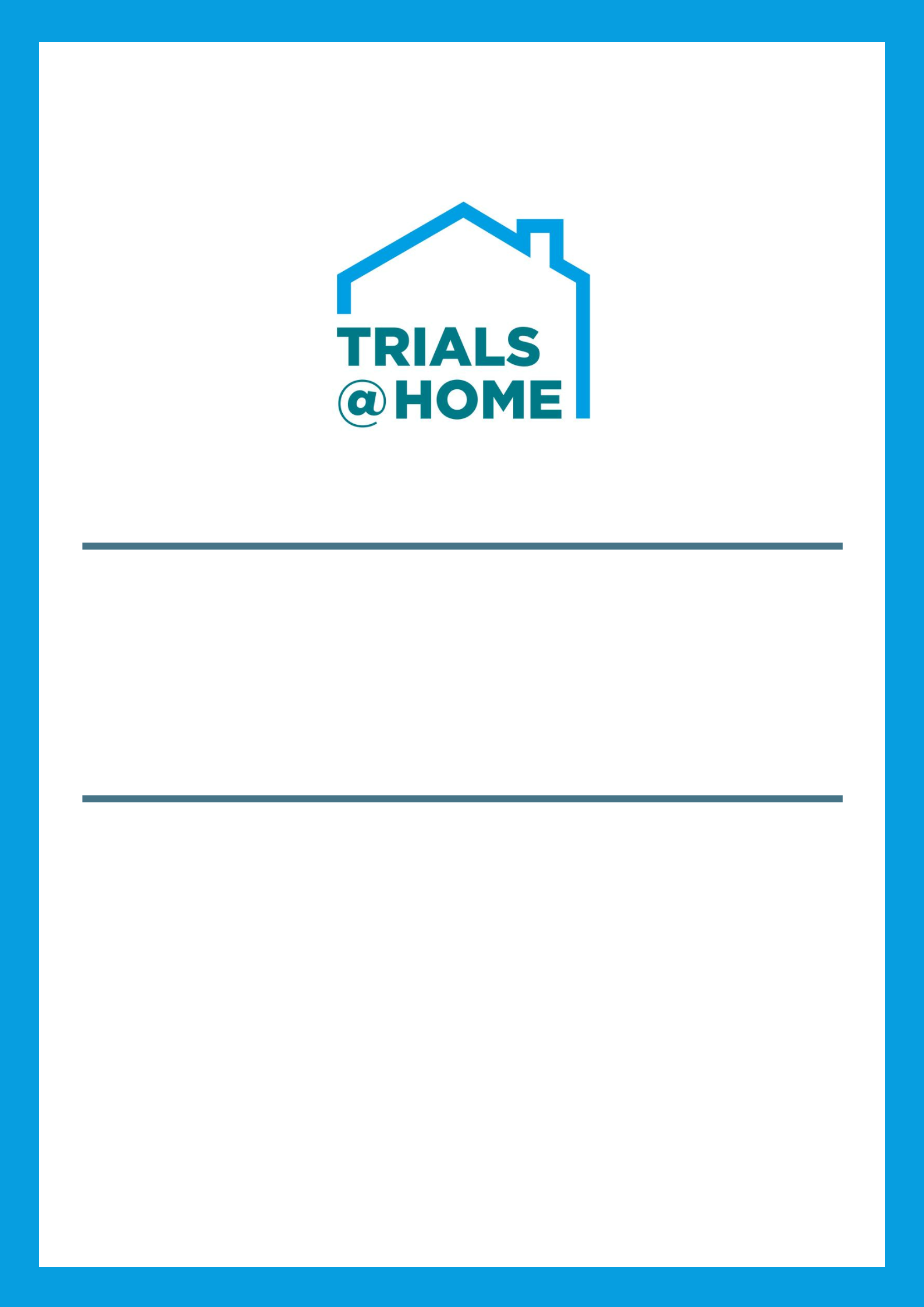


**June 2020**

**SURVEY**

The impact of COVID19 on
the remoteness of clinical trials

The research leading to these results has received support from the EU/EFPIA Innovative Medicines Initiative [2] Joint Undertaking (H2020-JTI-IMI2) Trials@Home grant n° 831458

**INTRODUCTION**

Thank you for taking time out of your schedule to fill in this survey.

This survey is sent out to all partners of the IMI Trials@Home consortium.

If you are not familiar with the specifics of the Trials@Home project, please find a short introduction here. The Trials@Home consortium will explore the opportunities of moving clinical trials from the traditional clinic setting to the participant’s immediate surroundings. These so-called Remote Decentralised Clinical Trials (RDCTs) make use of new – digital – innovations and enable participants to visit a clinical trial center less frequently, if at all. More information can be found at trialsathome.com

With the ongoing COVID19 crisis, RDCTs have become even more relevant and an acceleration in the adoption of remote approaches seems to be taking place. As a consortium, we have the unique opportunity to gain in-depth insights into the changes in clinical trials due to COVID19, and the associated challenges and opportunities.

In this survey we will ask about how COVID19 has impacted your trial operations, what changes have been made due to COVID19 and what the consequences of these changes were. We would also like to learn more about the problems and challenges you came across (or are encountering) as a consequence of the COVID19 crisis, and whether you envision to maintain the changes made in trials conduct after COVID19.

Your responses will help us tailor our project to new remote developments and optimize our recommendations and tools for the implementation of RDCTs across Europe.

There are no right or wrong answers to the questions, we are interested in your experiences during the COVID19 crisis. If your organization is not primarily responsible for trials conduct, please answer these questions as best as you can from your own perspective for the trials you are involved in.

Thank you again for your time, and should you have any questions or concerns regarding this survey, please reach out to Dr. Arnela Suman via [a.suman@umcutrecht.nl](mailto:a.suman@umcutrecht.nl).

*Data and Confidentiality*

*The University Medical Centre Utrecht (UMCU) will handle the information strictly confidential. The data will be stored within the UMCU, where access is limited to the Trials@Home study team. The information will not be available outside the UMCU. The link to the Trials@Home consortium and the type of organizations and companies will be mentioned in publications, however individuals and individual companies will not be named. Reported survey responses will always be anonymized and/or aggregated so that the information/examples cannot be traced back to individuals or companies.*

**QUESTIONS**

Date

Your organization’s usual trial role (e.g. industry sponsor, CRO, tech provider, site, etc.)

Your role within the organization, including key responsibilities

Please try to answer all the questions for your organization as a whole, if you are unable to do so, you can answer the questions for your unit or department. Please indicate if you answer these questions for your entire organization or for your unit/department:

*If you answer for your unit or department, please read unit/department where the survey says organization.*

How many clinical trials were ongoing within your organization prior to the COVID19 outbreak (rough estimate)?

*If this question is too difficult to answer, could you indicate whether this is in ones, tens or hundreds?*

1. What is the percentage of clinical trials within your organization that already incorporated remote aspects (see examples under question 3) before the COVID19 outbreak (rough estimate)?

None

1- 25%

26- 50%

51- 75%

76- 99%

100%

Don’t know *(Please try to make a rough estimate before choosing this option)*

If the answer to question 1 was none, please continue to question 4.

2. Of the trials that already incorporated remote aspects, how many of these trials were fully remote trials?

None

1- 25%

26- 50%

51- 75%

76- 99%

100%

Don’t know *(Please try to make a rough estimate before choosing this option)*

If the answer to question 2 was 100%, please continue to question 4.

3. What type of study activities were implemented in a remote fashion before the COVID19 outbreak?

Please think of all clinical trial phases and name as many aspects as you can think of (see examples in the table below this question). For ease of use, you can tick the boxes that apply, but this is not an exhaustive list, so please fill in your own answers in the textbox below.

| Operational feasibility assessment,  site selection/ qualification,  site initiation,  technology setup,  IMP supply. |
| --- |
| Participant outreach,  pre-screening,  participant education,  obtaining informed consent,  screening,  patient technology enablement. |
| Self-intervention and self-monitoring,  home health visits,  telemedicine visits,  clinic visits,  IMP adherence monitoring. |
| Decommissioning,  archiving. |
| Data collection,  Management of study-generated data,  gathering and management of real-life data,  clinical data repository management,  data reconciliation and query management,  source document verification. |
| Clinical monitoring,  performance monitoring,  inspection facilitation,  system approval facilitation,  safety (data) management. |
| Social listening and patient landscape analysis,  provide updates to patients throughout the trial,  provide patient recruitment and retention incentives,  patient concierge service,  introducing behavioral incentives,  patient-HCP interaction and communication,  provide direct patient messaging,  patient social community establishment. |

4. How did the COVID19 outbreak impact the conduct of the clinical trials within your organization?

1. Trials were halted . If yes, what percentage of trials were halted (rough estimate)?

1- 25%

26- 50%

51- 75%

76- 99%

100%

Don’t know *(Please try to make a rough estimate before choosing this option)*

1. Trials continued without modifications . If yes, what percentage of trials could continue as usual (rough estimate)?

1- 25%

26- 50%

51- 75%

76- 99%

100%

Don’t know *(Please try to make a rough estimate before choosing this option)*

1. Trials continued with modifications . If yes, what percentage of trials could continue with modifications (rough estimate)?

1- 25%

26- 50%

51- 75%

76- 99%

100%

Don’t know *(Please try to make a rough estimate before choosing this option)*

If you did not have to put trials to a halt, please continue to question 6.

5. If trials were halted, what were the main reasons for the discontinuation?

If none of the trials continued without modifications, please continue to question 7.

6. For the trials that continued without modifications, what were the main characteristics of these trials that allowed them to continue without modifications?

If none of the trials continued with modifications, please continue to question 10.

7. For the trials that continued with modifications, in which therapeutic areas did these trials take place?

8. What changes were implemented for ongoing trials to address the issue of the COVID19 outbreak?

1. Implement some remote aspects . If yes, in what percentage of trials that continued with modifications were remote aspects implemented (rough estimate)?

1- 25%

26- 50%

51- 75%

76- 99%

100%

Don’t know *(Please try to make a rough estimate before choosing this option)*

1. Changed to fully remote trials . If yes, what percentage of trials that continued with modifications changed to completely virtual trials (rough estimate)?

1- 25%

26- 50%

51- 75%

76- 99%

100%

Don’t know *(Please try to make a rough estimate before choosing this option)*

1. Other changes , namely      . What percentage of trials that continued with modifications implemented these changes (rough estimate)?

1- 25%

26- 50%

51- 75%

76- 99%

100%

Don’t know *(Please try to make a rough estimate before choosing this option)*

In case of multiple changes, please fill them in below, together with a rough percentage.

If no remote aspects were implemented in ongoing trials, please continue to question 10.

9. For the trials that implemented remote aspects in order to be able to continue, what type of study activities were implemented in a remote fashion?

Please think of all clinical trial phases and name as many aspects as you can think of (see examples in the table below this question). For ease of use you can tick the boxes that apply, but this is not an exhaustive list, so please fill in your own answers in the textbox below.

| Operational feasibility assessment,  site selection/ qualification,  site initiation,  technology setup,  IMP supply. |
| --- |
| Participant outreach,  pre-screening,  participant education,  obtaining informed consent,  screening,  patient technology enablement. |
| Self-intervention and self-monitoring,  home health visits,  telemedicine visits,  clinic visits,  IMP adherence monitoring. |
| Decommissioning,  archiving. |
| Data collection,  Management of study-generated data,  gathering and management of real-life data,  clinical data repository management,  data reconciliation and query management,  source document verification. |
| Clinical monitoring,  performance monitoring,  inspection facilitation,  system approval facilitation,  safety (data) management. |
| Social listening and patient landscape analysis,  provide updates to patients throughout the trial,  provide patient recruitment and retention incentives,  patient concierge service,  introducing behavioral incentives,  patient-HCP interaction and communication,  provide direct patient messaging,  patient social community establishment. |

10. What were the greatest challenges for conducting clinical trials since the COVID19 outbreak?

11. Which of the implemented remote aspects are working well and why?

12. Which of the implemented remote aspects are not working well and why?

13. Which of the implemented remote aspects do you expect/plan to be maintained within the current trials after the COVID19 crisis?

14. Are there any permanent changes to trial conduct being planned for future trials?

If yes, please specify which changes and to what extent these include remote approaches.

15. What are the most important lessons that your organization is learning as a result of COVID19 regarding clinical trial conduct?

**This is the end of the survey. Thank you very much for your time. The aggregated results will be reported back to the consortium. We might come back to you for more in-depth follow-up questions.**
